# Optimization of Elderly Influenza and Pneumococcal Immunization Programs in Beijing, China Using Health Economic Evaluations: A Modeling Study

**DOI:** 10.3390/vaccines11010161

**Published:** 2023-01-11

**Authors:** Zhenfei Pi, Kiyoshi Aoyagi, Kazuhiko Arima, Xiaoliang Wu, Zhaojia Ye, Yawen Jiang

**Affiliations:** 1 Department of Public Health, Nagasaki University Graduate School of Biomedical Sciences, 1-12-4 Sakamoto, Nagasaki 852-8523, Japan; 2 Shenzhen Center for Disease Control and Prevention, Shenzhen 518055, China; 3 School of Public Health (Shenzhen), Sun Yat-sen University, Room 533, West Wing of Medical Complex #1, 66 Gongchang Road, Guangming District, Shenzhen 518107, China

**Keywords:** cost-effectiveness, influenza, pneumococcal, vaccine

## Abstract

(1) Background: Currently, residents ≥ 60 and ≥65 years old in Beijing, China, are eligible for free influenza and pneumococcal polysaccharide vaccines (PPSV23), respectively. The present study aimed to assess the cost-effectiveness of current and alternative strategies of dual influenza and PPSV23 vaccination among the elderly in Beijing. (2) Methods: We developed a Markov state-transition model to compare the costs and the quality-adjusted life years (QALYs) associated with four influenza and PPSV23 vaccination strategies among the elderly in Beijing. The strategies were as follows: (1) no vaccination; (2) only flu vaccine for people ≥ 60 years old; (3) flu vaccine for people ≥ 60 years old and PPSV23 for people ≥ 65 years old; and (4) dual influenza vaccines and PPSV23 for people ≥ 60 years old. Incremental costs and QALYs were quantified to determine the optimal option. If dominant strategies emerged, the Chinese gross domestic product per capita in 2021 (80,976 CNY) was used as the willingness-to-pay (WTP) threshold to covert QALYs into the monetary equivalent. (3) Results: The current program saved costs and increased QALYs compared to no vaccination or flu vaccine-only strategies. However, extending free PPSV23 to people ≥ 60 years old saved 0.35 CNY additionally while increasing QALYs marginally compared with the current policy. Results were robust in all sensitivity analyses. (4) Conclusion: Beijing’s current dual influenza and pneumococcal vaccination program was cost-effective among the elderly compared with the preceding policies of no vaccination and flu-only immunization programs. However, the program can further save money while enhancing the population health by extending PPSV23 to all people ≥ 60 years old.

## 1. Introduction

Influenza and pneumonia both rank among the top respiratory causes of mortality and morbidity in China, the burden of which is disproportionately high among the elderly [1,2,3]. Recent estimates suggest that influenza cause about 90,000 excess deaths annually in China, among which people aged 60 years and older are the most represented age group [1]. Pneumococcal infection is not only a major standalone cause of mortality, but also a relatively common influenza-related complication that exacerbates the prognosis of influenza infection [3,4]. To date, influenza and pneumococcal vaccines remain two of the most potent tools against both types of infections [5,6]. However, the uptake rates of influenza and pneumococcal vaccines among the Chinese elderly have been suboptimal [7,8], which is largely attributable to an absence of national immunization programs for both vaccines [9].

To turn the tide against low vaccination rates, a fraction of Chinese cities has initiated local efforts to immunize their most vulnerable population, a leading example of which is free influenza and pneumococcal polysaccharide vaccines (PPSV23) for seniors in Beijing [9,10]. Specifically, free inactivated influenza vaccines have been provided to seniors aged ≥ 60 years old since 2007, and free PPSV23 have been provided to people aged ≥ 65 years and older since 2018 [9,10]. While the policy on influenza immunization is compliant with the recommendations of the China National Immunization Advisory Committee and the Chinese Medical Association Gerontology Branch, the policy on using PPSV23 is more restrictive than the recommendations of the 60-year-old starting age [11,12,13].

Intuitively, expanding the eligible population increases upfront investment. However, rational planning of age-based immunization strategies optimizes resource allocation by improving health outcomes while reducing costs of medical occurrences [14,15]. In fact, providing both influenza vaccines and PPSV23 to seniors aged 60 years and above free of charge has been shown to be cost-effective in other Chinese cities [16]. Accordingly, it is also important to determine whether the current Beijing immunization programs against influenza and pneumococcal pneumonia among different age groups represent under-use or abuse of resources. To that end, we conducted model-based cost-effectiveness analyses of four strategies to examine whether public-funded influenza and PPSV23 immunization programs among Beijing seniors can be improved. The present findings will help inform the planning of municipal public health strategies in Beijing and other Chinese cities.

## 2. Methods

### 2.1. Model Overview

**Figure 1:**
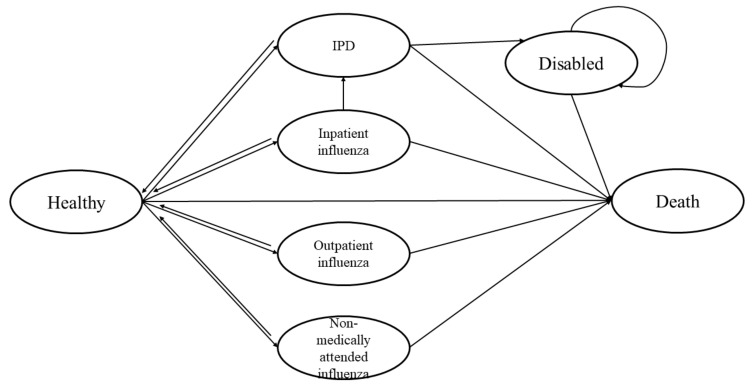
The structure of the Markov model. IPD, invasive pneumococcal disease.

We programmed a Markov state-transition model that progressed with weekly cycles using Microsoft Excel 2019 (Microsoft Corporation, Redmond, WA, USA) and VBA. Disease incidence, deaths, costs, and quality-adjusted life years (QALYs) associated with alternative vaccination strategies among a hypothetical cohort of elderly people in Beijing were accrued via the simulation process of the model. The life expectancy at birth in China is roughly 78 years. However, stopping the simulation at the age of 78 years would only capture the final outcomes of about half of the cohort. To fully capture the costs and QALYs, the time horizon was extended from the start age until either death or 100 years old to comply with recommendations [17]. The model was populated with a hypothetical cohort of 100,000 individuals. It is noteworthy that the intervention strategies (introduced in the following subsection) involved age-based eligibility. To allow age-dependent vaccination strategies, the model simulated two cohorts in parallel. Namely, they were the 60–64-year-old cohort and the ≥ 65-year-old cohort. The start age of 60–64 years old was 62 years, and the start age of the ≥ 65-year-old cohort was 74 years, each of which depended on the mean age of the corresponding cohort to reflect the average profiles [18]. The costs and the QALYs of the two cohorts were then summed up using their proportions out of the entire ≥ 60-year-old population as weights. Seven health states were defined to capture the different severity levels caused by clinical manifestations of influenza and pneumococcal infections. Namely, they were healthy, non-medically attended flu, outpatient flu, flu hospitalization, invasive pneumococcal diseases (IPD), disabled, and death (Figure 1). All individuals started in a healthy state. Throughout the cycles, individuals experienced the risks of influenza infection, IPD, disability, and death. Based on the literature evidence, we assumed that PPSV23 reduced the risk of IPD, but not non-invasive pneumonia. Moreover, three distinct influenza-related health states were defined to reflect different severities. Namely, they were skipped medical visits (self-treatment), sought outpatient care and sustained a hospitalization [19,20]. After hospitalization due to influenza, patients could develop IPD as a complication in our model. Following the development of IPD, patients had the risk of becoming disabled, which was an irreversible state such that patients could only either stay as disabled or become diseased afterwards. By contrast, patients in other influenza- and IPD-related states could recover completely and return to the healthy state.

### 2.2. Vaccination Strategies

In the present analysis, four strategies were evaluated: (1) no vaccination for either pathogen; (2) only influenza vaccine for people aged ≥ 60 years old; (3) the current policy of influenza vaccine for people aged ≥ 60 years old and PPSV23 for people aged ≥ 65 years old; and (4) dual influenza and PPSV23 for people aged ≥ 60 years old. In line with the average starting time of seasonal influenza epidemics, individuals received the influenza vaccine in the 40th week of each year in strategies 2–4. In addition, eligible individuals received PPSV23 once at the starting age in strategies 3 and 4. Based on previous analyses of Beijing’s free influenza vaccination program, 19% of elderly people received the influenza vaccine in the model [21]. Similarly, 23% of seniors received PPSV23 based on the literature evidence from Shanghai, which had a free PPSV23 immunization program for the elderly just like Beijing [22].

### 2.3. Epidemiological and Clinical Inputs

Table 1 lists the inputs of the model. Whenever possible, parameter values were sourced from country-specific and local data in the published literature. Based on a meta-analysis estimating the annual attack rate of seasonal influenza, the annual incidence of influenza among unvaccinated persons was set at 0.107/person-year [23]. To incorporate the seasonal activity of influenza, we modeled the incidence of influenza ($\beta_{t}$) as a sinusoidal function over time [24]. Specifically, the formula$\;\beta_{t}=\beta_{1}\left[1+\cos\left(2\pi t\right)\right]\;$was used, where$\;\beta_{1}\;$represents the baseline incidence of influenza and *t* is the time of the current weekly cycle. The baseline IPD incidence among unvaccinated individuals was obtained from a Hong Kong study due to the absence of the corresponding data for people in mainland China [25]. The reported annual incidence rate of IPD in those aged ≥ 65 years was 8.3 per 100,000 person-years, which was transformed into probability using the equation $P_{year}=1-\;\exp\left(-r_{year}\right)$, where $P_{year}\;$and$\;r_{year}$ are the probability and incidence rate, respectively [26]. To accommodate annual probabilities to the weekly cycle of the current model, we used the declining exponential approximation of life expectancy (DEALE) method for data conversion [27]. Formally, the calculation was:$\;r_{week}=-\left(1/52\right)\;\ln\left(1-P_{year}\right)$, $P_{week}=1-\exp\left(-r_{week}\right)$, where $\;r_{week}$ and $P_{week}$ are the weekly incidence rate and the weekly probability, respectively.

The extent to which influenza vaccines matched circulating strains leads to a varying influenza vaccine effectiveness (VE) across flu seasons, so it was necessary to use representative figures of influenza VE that reflect the overall protective effect of influenza vaccines. To that end, we used data from a recent meta-analysis for the average profile of influenza VE, which suggested that the influenza vaccine had a matched VE of 44.38% and a mismatched VE of 20.00% among the elderly [28,45]. In the baseline analysis, we used the mean of matched and mismatched VEs. To explore the impacts of the uncertainty in this parameter, we explored a range of VEs from 20 to 44.38% in sensitivity analyses. Similarly, we sourced data from a systematic review for the VE of PPSV23 against IPD [29]. On top of the VE of PPSV23, we also applied a PPSV23 serotype coverage of 77% according to a study in Hong Kong, China [30]. The probability of IPD among vaccinated individuals was calculated as: probability among unvaccinated person × [1 − (VE × serotype coverage × vaccination rate)].

In the absence of data sources from Beijing and elsewhere in China, the fractions of outpatient and inpatient influenza patients out of all influenza-positive individuals were obtained from a retrospective study in the US [31]. In addition, the risk of developing IPD was 10% for hospitalized influenza patients according to the literature [19]. More, it is noteworthy that estimates on the probability of becoming disabled due to IPD was not available in the literature, and was proxied using the fraction of meningitis that would lead to disability [32]. Finally, the probabilities of returning to the healthy state from influenza- and IPD-related hospitalizations were calculated using the ratio of the length of the modeling cycle to the average length of hospital stays.

General population mortality rates by 5-year age interval were obtained from the World Health Organization (WHO) life tables and converted into weekly probabilities [26,27,33]. In addition, the probability of in-hospital mortality due to influenza was derived from the literature [34]. Moreover, the probability of dying from IPD was retrieved from an observational study on pneumococcal diseases in Taiwan, China [35]. Finally, we extracted data on disability-related excess mortality from a published economic analysis [19].

### 2.4. Health State Utilities

Health outcomes in the present analysis were quantified using QALYs, which necessitated the inputs of health-state utility values (HSUVs). Of note, elderly people are not in a perfect health state even without influenza and IPD. To reflect such background health attrition, we extracted age-specific health utility scores from the 2008 National Health Services Survey in China that collected EuroQol-5 Dimension Questionnaire (EQ-5D) responses [36]. The HSUVs of outpatient and hospitalized influenza patients were based on a study that surveyed the disease states of influenza patients in China using EQ-5D [37]. For non-medically attended influenza patients, we assumed a utility score decrement of 0.05. In addition, the utility scores of IPD and disabled patients were derived from economic evaluations in the literature [19,38].

QALY increment can be decomposed into two parts, which are sickness days and life year lost, respectively. Therefore, information on the duration of the illness was also required. In a Chinese study, the average duration of influenza episodes that require outpatient visits was 6.2 days [37]. The inpatient length of stay associated with influenza was taken from a survey that estimated the burden of influenza in China [39]. In addition, the average length of hospital stay for IPD patients was 12 days according to an estimation in Beijing [25,40]. Finally, the QALY loss attributable to premature mortality were calculated based on the life expectancy and the age-specific utility scores of the general elderly population in China. In the present analysis, outcomes were discounted at an annual rate of 5% based on the Chinese guidelines for pharmacoeconomic evaluations [33,44].

### 2.5. Costs

Incorporating both direct and indirect burden, the present analysis took a societal perspective to analyze costs and outputs. Direct costs including vaccine acquisition and injection costs, medical costs for influenza-related illness, and IPD hospitalization costs. On top of that, indirect costs due to productivity loss of caregivers during hospitalization and disability episodes were also accrued. All costs were inflated to 2020 CNY using the healthcare component consumer price index (CPI) in China [46]. The prices of inactivated trivalent influenza vaccines (28 CNY/dose) and PPSV23 (200 CNY/dose) were sourced from an announcement on the purchase of vaccines by the Beijing government and a website collecting pharmaceutical bidding prices in China [41,42]. The costs of non-medically attended influenza, which mainly pertained to over-the-counter (OTC) drugs, were estimated to be 100 CNY based on the average costs of common OTC cold medicine and antipyretics [42]. Medical costs of influenza outpatient visits, influenza hospitalizations, and IPD episodes were taken from investigations on influenza and pneumonia in Beijing [21,40]. To be compatible with the model, influenza and IPD hospitalization costs were transformed to weekly equivalents using the corresponding lengths of stay. To calculate indirect costs, we used the human capital approach to quantify productivity loss in the model. Of note, no productivity loss due to influenza- and IPD-related illness or deaths for those aged 60 years and older was counted, since the legal retirement age in China is 60 years. In contrast, productivity loss of caregiving time devoted to hospitalized and disabled patients was calculated. Specifically, we assumed that disabled patients consumed two hours of care per day whereas each hospitalized patient required full-day long company. To monetize the caregiving time, we used the average salary in Beijing. According to the Beijing Bureau of Statistics, the average daily compensation rate in 2020 was 656.08 CNY [43]. In the present study, an average person was assumed to work 22 days per month and 8 h per day.

### 2.6. Decision Rules

Despite the intuitive attempt, it was inappropriate to calculate incremental cost-effectiveness ratios (ICERs) of all strategies in relation to a common reference strategy to the extent that the current decision problem pertains to a comparison of multiple mutually exclusive strategies [17]. Instead, we followed the decision rule of eliminating dominated and extensively dominated strategies first and then calculating ICERs of remaining strategies in sequence for the rest of the strategies. The willingness-to-pay (WTP) threshold of the gross domestic product (GDP) per capita was then used to determine the optimal strategy. If all but one strategy was dominated or extensively dominated, then net monetary benefit (NMB) was calculated. Specifically, the following steps were taken to determine the optimal strategy:

1. Simulate costs and QALYs associated with each strategy;
2. Sequence the strategies by costs in increasing order;
3. Determine whether each strategy iss dominated by the next strategy starting from the least expensive strategy. Repeat this step, until there are no dominated strategies;
4. Determine whether each strategy (e.g., strategy k) is extensively dominated by the next strategy by comparing the ICER of the next strategy (strategy k + 1) in relation to the previous strategy (strategy k − 1) to that of the running ICER (ICERs of strategy k vs. strategy k − 1). If the ICER of strategy k + 1 vs. k − 1 is smaller than the running ICER, then strategy k is extensively dominated. This is repeated, until there are no extensively dominated strategies;
5. Identify the strategy with the ICER is immediately below the WTP threshold as the optimal strategy;
6. If ICERs are not applicable, the NMB of the optimal strategy in reference to the current policy (strategy 3) is calculated.

### 2.7. Sensitivity Analyses

One-way sensitivity analyses (OWSA) were conducted by varying all probabilities, effectiveness, costs, utility, and hospitalization duration data. All parameters were adjusted upward and downward by 20% of their original values in OWSA except for utilities, which were adjusted by 10% due to unrealistic numbers caused by a 20% variation. Probabilistic sensitivity analyses (PSA) were performed by simultaneously resampling parameters from pre-specific distributions of input values. The resampling was repeated 1000 times. In the PSA, costs and other healthcare resource utilization parameters were defined as gamma distributions, whereas probabilities, rates, ratios, and utilities were defined as beta distributions, the standard errors of all of which were 0.1 times of the corresponding mean values.

## 3. Results

The base-case results of the analysis are displayed in Table 2. The strategies of no vaccination (strategy 1), influenza vaccine for people aged ≥ 60 years (strategy 2), influenza vaccine for people aged ≥ 60 years, PPSV23 for people aged ≥ 65 years (strategy 3), and both vaccines for people aged ≥ 60 years (strategy 4) were associated with 2746.1 CNY, 2589.2 CNY, 2585.0 CNY, and 2584.6 CNY of average total costs, respectively. Based on these results, each more inclusive strategy (the strategy that vaccinates more individuals) actually saved costs compared to the less inclusive strategy. Accordingly, strategy 4 was the least costly strategy. On top of that, strategy 4 was also the most effective option by generating 8.905139 QALYs for an average person. By contrast, strategies 1–3 were associated with health outputs of 8.900666 QALYs, 8.905088 QALYs, and 8.905136 QALYs, respectively. Similar to the results of costs, each more inclusive strategy increased QALYs compared to the less inclusive strategy. Taken together, strategies 1–4 were descending in costs while ascending in QALYs. In other words, more generous strategies always saved money and improved health. Therefore, strategies 1–3 were dominated by more inclusive but less expensive strategies, enabling strategy 4 to be the optimal strategy.

**Figure 2:**
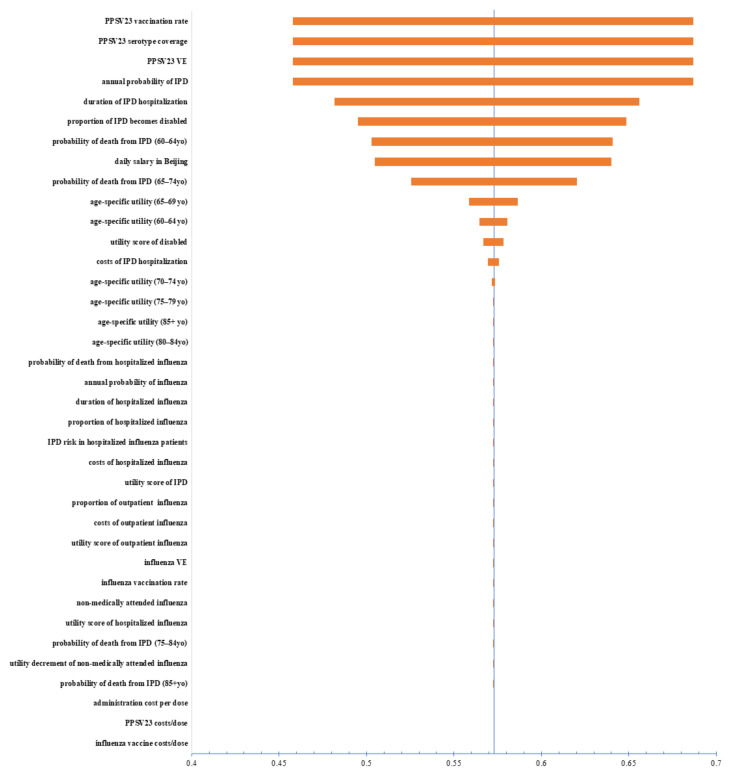
The NMB changes of strategy 4 compared with those of the current policy (strategy 3) in OWSA. PPSV23, pneumococcal polysaccharide vaccines; IPD, invasive pneumococcal disease; VE, vaccine effectiveness; YO, years old.

**Figure 3:**
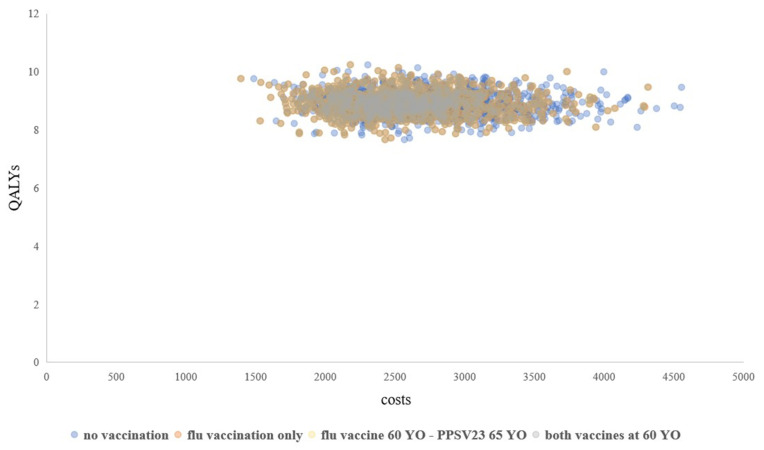
The scatterplots of QALYs against costs associated with the four strategies from the PSA. QALY, quality-adjusted life year; YO, years old.

In the OWSA, strategy 4 remained the optimal strategy when parameters were adjusted upward and downward, the results of which were not shown since they were not informative. Alternatively, the NMB changes of strategy 4 compared with those of the current policy (strategy 3) in OWSA are shown in Figure 2. Strategy 4 consistently resulted in a net benefit compared with the current policy. Of note, the NMB was relatively sensitive to the uptake rate of PPSV23 of the target population, the serotype coverage of PPSV23, the VE of PPSV23, and the annual probability of contracting IPD. In fact, the variations of these four parameters changed the NMB by the same magnitude. This was because all four parameters collinearly affected the incidence of IPD for those who were vaccinated with PPSV23. However, none of the changes was sufficiently substantial to nullify the base-case result. Also suppressed in our exhibits are the cost-effectiveness acceptability curves from PSA, which would have been straight lines for all strategies had they been shown because strategy 4 remained the dominant strategy in the simulations. The scatterplots of QALYs against costs associated with the four strategies from the PSA are depicted in Figure 3. Since the *x*- and *y*-axes were not incremental values, the scatterplots in Figure 3 did not have an intuitive interpretation as in a two-strategy comparison. However, the dispersion of the scatterplots may still be informative. Specifically, the dominance of strategy 4 is not evident at the first sight, but the relative scarcity of strategy 4 data points at the far right of the plane suggests that the PSA results of strategy 4 were less spread-out from the base case than other strategies. Given the dominance of strategy 4 in the base case, the probability that strategy 4 remains dominant in the simulations is also high. By contrast, the scatterplots of the other strategies were less condensed and more frequently distributed at the far-right section of the plane, thereby having large chances of producing less QALYs but incurring higher costs than strategy 4.

## 4. Discussion

In the present analysis, we conducted economic evaluations of the current policy and three alternative programs of influenza and PPSV23 immunization policies among seniors in Beijing, China. The results suggest that the current policy in Beijing, although cost-saving compared with less generous strategies, can be further enhanced by expanding PPSV23 coverage to all people aged 60 years and older. Such a strategy creates additional health benefits for the population aside from being cost-saving. In fact, the total cost saving of PPSV23 coverage expansion compared with that of the current strategy can amount to over 1.7 million CNY with a population health increment of 13 QALYs if scaled up to the entire older population of Beijing.

It has been shown that the dual vaccination of influenza vaccine and PPSV23 reduces the risk of hospitalization among 60 years and older Chinese people and is cost-effective compared with no vaccination in other cities [16,47]. However, immunization programs in many Chinese cities are not necessarily in line with evidence. The findings of the current analysis enclose unambiguous messages for public health policies. First, it is advisable to immunize those aged 60–64 years old with PPSV23 in addition to the elderly population of ≥65 years old who are already eligible while maintaining the current influenza vaccination policy. Expanding protection against respiratory pathogens among the elderly is a critical task. Although COVID-19 may seem to be a prima facie threat of respiratory infectious diseases at the current stage, conventional pathogens should not be neglected. Elderly people are at disproportionately high risks of severe complications after infections of respiratory pathogens. In the meantime, natural immunity against influenza and pneumonia may have been compromised to a certain degree due to their suppression in the past two winters. Second, but less explicitly, the incremental uptake of both influenza vaccine and PPSV23 among the elderly population is cost-saving. As such, the priority of vaccine type is not necessarily of foremost concern for cities with similar socioeconomic development as Beijing that wish to engage in immunization programs of the two vaccines.

Whereas the absolute values of QALY gain per capita in the present analysis may seem trivial compared with the treatments of cancer and other end-stage diseases, such interpretations may be misleading. From the perspective of health economics, it is the per-dollar payoff that matters, since it is comparable across disease areas, treatments, and strategies. By contrast, the magnitude of QALY differences attributable to interventions is not necessarily comparable across diseases. Accordingly, the determination of the optimal strategy should strictly abide by the pre-specified decision rules.

Cost-effectiveness studies on dual influenza and pneumococcal immunization programs usually choose to compare two alternatives [19,48]. Such comparisons would only allow the identification of a cost-effective option among very limited choices. To explore more options, multi-strategy economic evaluations are necessary. In the present analysis, we conducted a multi-strategy comparison, such that it was possible to optimize the strategy instead of only confirming the cost-effectiveness of the current program.

The findings of the current analysis should be interpreted with several caveats. First, some of the epidemiological and vaccine uptake rate parameters were not sourced from the local data of Beijing, the availability of which is limited. Second, population-level natural immunity from previous influenza infections was not accounted for. However, the impact of omitting this item should not be overstated, since natural immunity may have reached a minimal level due to the suppression of flu during the COVID-19 pandemic [49]. Third, the present analysis did not engage a dynamic transmission model to consider vaccination-induced herd immunity. Therefore, the current results may represent underestimates of true benefits. Of note, static models for the cost-effectiveness analysis of influenza and pneumococcal vaccination programs are among the recommendations by the WHO guideline for standardization of economic evaluations of immunization programs [50]. Finally, we did not analyze the use of 13-valent pneumococcal conjugate vaccines (PCV13) among older adults, since it is only indicated for children in China. PCV13 is approved for people ages 50 years and older to prevent pneumonia and invasive disease in many Western countries but has yet to receive approvals for the same age groups in China. Future economic analyses of vaccination strategies should factor this new type of pneumococcal vaccine into the equation if its use is expanded. Also unavailable to Chinese population now are 15-valent and 20-valent pneumococcal vaccines (PCV15 and PCV20). Insights into the potential economic profiles of the innovative vaccines in future will help to shape rational immunization policies. Despite the limitations summarized above, the economically preferable profile of the most generous option among the investigated strategies is unlikely to be undermined due to the dominance of this option and the robustness of the results. Hence, our analysis provides important information to the evidence base of the planning of immunization programs in Beijing and probably elsewhere in China.

## 5. Conclusions

The current policy of providing free influenza vaccine for people aged ≥ 60 years and PPSV23 for people aged ≥ 65 years in Beijing saved money and reduced disease burden compared with preceding policies that were less generous. However, providing PPSV23 coverage to all people aged 60 years and older can further reduce total healthcare costs and improve population health. As such, the expansion of the free PPSV23 target population among the older population in Beijing is recommended.

## Figures and Tables

**Table 1:** Inputs of the Markov model.

Inputs	Base-Case Value	Value Change for OWSA	Distribution for PSA	Reference
**Clinical inputs**				
Influenza vaccination rate	0.19	±20%	beta	[21]
Annual probability of influenza infection in the unvaccinated elderly	0.23	±20%	beta	[22]
Annual probability of IPD in the unvaccinated elderly per 100,000	8.3	±20%	beta	[25]
Influenza vaccine effectiveness	32%	±20%	beta	[28]
PPSV23 effectiveness	50%	±20%	beta	[29]
PPSV23 serotype coverage	0.77	±20%	beta	[30]
Proportion of non-medically attended influenza	0.34	-	-	Estimated
Proportion of outpatient influenza	0.62	±20%	beta	[31]
Proportion of hospitalized influenza	0.04	±20%	beta	[31]
Probability of hospitalized influenza patients to develop IPD	0.10	±20%	beta	[19]
Proportion of IPD patients to become disabled	0.07	±20%	beta	[32]
Probability of recovery from influenza hospitalization	0.78	-	-	Estimated
Probability of recovery from IPD	0.54	-	-	Estimated
Annual background mortality rate death per 1000 (60–64 years old)	2.0	±20%	beta	[33]
Annual probability of natural death per 1000 (70–74 years old)	5.9	±20%	beta	[33]
Probability of death from hospitalized influenza	0.12	±20%	beta	[34]
Probability of death from IPD	0.16	±20%	beta	[35]
Mortality ratio of the disabled	1.1		-	[19]
**Utility scores**				
60–64 years old	0.74	±10%	beta	[36]
65–69 years old	0.71	±10%	beta	
70–74 years old	0.69	±10%	beta	
75–79 years old	0.68	±10%	beta	
80–84 years old	0.66	±10%	beta	
Utility decrement of non-medically attended influenza	0.05	±10%	beta	Assumed
Utility score of outpatient influenza	0.61	±10%	beta	[37]
Utility score of hospitalized influenza	0.56	±10%	beta	[37]
Utility score of IPD	0.20	±10%	beta	[38]
Utility score of disabled	0.40	±10%	beta	[19]
**Duration**				
Length of non-medical/outpatient influenza (days)	6.2	-	-	[37]
Length of hospitalized influenza (days)	9	±20%	gamma	[39]
Length of IPD (days)	12	±20%	gamma	[40]
**Costs (2020 CNY)**				
Price of influenza vaccine per dose	28	±20%	gamma	[41]
Price of PPSV23 per dose	200	±20%	gamma	[42]
Vaccine administration costs per dose	25	±20%	gamma	[21]
Non-medically attended influenza cost	100	±20%	gamma	Estimated
Outpatient influenza cost	421	±20%	gamma	[21]
Hospitalized influenza cost	15,870	±20%	gamma	[21]
IPD hospitalization cost	12,460	±20%	gamma	[40]
Daily labor compensation rate in Beijing	656	±20%	gamma	[43]
**Average life expectancy**				
60–64 years old	21	-	-	[33]
70–74 years old	13	-	-	[33]
**Annual discount rate**	5%	-	-	[44]

Abbreviations: OWSA, one-way sensitivity analysis; PSA, probabilistic sensitivity analysis; IPD, invasive pneumococcal disease; PPSV23, pneumococcal polysaccharide vaccine.

**Table 2:** Base-case results.

Strategy NO.	Strategy	Costs (CNY)	QALYs	ICER	NMB (CNY)
1	No vaccination	2746.1	8.900666	Dominated *	NA
2	Flu vaccine for people ≥ 60 YO	2589.2	8.905088	Dominated *	NA
3	Flu vaccine for people ≥ 60 YO and PPSV23 for people ≥ 65 YO	2585.0	8.905136	Dominated *	Reference strategy
4	Both vaccines for people ≥ 60 YO	2584.6	8.905139	Optimal	0.57

Abbreviations: YO, years old. * ICER is not defined for a dominated strategy.

## Data Availability

Data are available from the correspondent upon reasonable requests.
